# Seizure in HIV-infected patients: clinical presentation, cause and treatment outcome in Ethiopia—a retrospective study

**DOI:** 10.1186/s12879-021-06497-7

**Published:** 2021-08-10

**Authors:** Amanuel Amare

**Affiliations:** Department of Neurology, Addis Ababa University, Addis Ababa, Ethiopia

**Keywords:** Africa, HIV, Seizure, Epilepsy, Antiretroviral therapy, Antiepileptic drugs

## Abstract

**Background:**

The estimated number of adult patients living with HIV infection in Ethiopia in 2012 was approximately 800,000. Seizure occurs in 2 to 3% and 6.1% to 34.3% in patients with HIV infection and patients with neurological complications of HIV infection, respectively. Studies on HIV infection and seizure are rare in Ethiopia. The purpose of this study was to assess clinical presentation, cause and treatment outcome of patients with HIV infection presented with seizure.

**Methods:**

In this retrospective study, patients aged ≥ 13 years with HIV infection presented with seizure were included. Medical records were reviewed and demographic and clinical data were collected.

**Results:**

Records of 146 patients were analysed. Males were 55.5% and the mean age was 34 years. The diagnosis of HIV infection was made after current hospital admission in 69% of patients. Almost all patients (98.6%) had stage 4 HIV infection with very low CD4 count (mean = 77/mm^3^). In almost all patients seizure was a recent onset at current admission; either it started after admission (42.5%) or within 3 months prior to admission (52.5%). The types of seizures were: generalized tonic–clonic seizure [GTCS] (69.2%), focal motor with secondarily generalization [FMWSG] (19.9%) and simple focal motor (11%). The common causes of seizure were: cerebral toxoplasmosis (46%), tuberculous meningitis (35.6%) and cryptococcal meningitis (13.7%). Case-fatality was 53% and predictors of mortality were: seizure started after admission, change in mentation and comatose at initial evaluation.

**Conclusions:**

Most patients had stage 4 HIV infection with very low CD4 count and a recent onset seizure which started within 3 months at initial evaluation. GTCS was the commonest seizure type and most causes of seizure were central nervous system opportunistic infections. The case-fatality was high and change in sensorium was an independent predictor of mortality. To prevent the high mortality and morbidity prevention of HIV infection, early diagnosis and treatment, improving diagnostic facilities and access to non-enzyme inducing antiepileptic drugs are recommended.

## Background

The estimated number of adult patients living with HIV infection in Ethiopia in 2012 was approximately 800,000 [1]. Neurologic complications occur in 39 to 75% of the patients with HIV infection [2, 3]. The incidence of seizure in HIV- infected patients varies from 2 to 3% in prospective studies [3, 4]. The incidence of seizure in HIV infected patients in retrospective studies varies from 6.1 to 11% [5, 6] and 19.8–34.3% [7, 8] in HIV infected patients with neurological complications. Seizure occurs principally in patients with advanced HIV disease [4, 9].

The causes of seizure in HIV-infected patients include mass lesion, meningitis, HIV-encephalopathy, drug toxicity, metabolic derangements and idiopathic which might include incidental epilepsy or seizure due to the HIV itself [3–14]. Primarily generalized seizure was the most common type in most studies [4–7, 9, 12, 13].

In a retrospective study which assessed the pattern and treatment outcome of HIV-infected patients with neurological complications seizure was an independent predictor of mortality [8].

The aims of this study were to assess the clinical presentation, cause and treatment outcome of patients with HIV-infection presented with seizure to Tikur Anbessa Specialized Hospital (TASH), the largest teaching hospital of Addis Ababa University in Ethiopia.

## Methods

HIV infected patients aged ≥ 13 years with seizure who were admitted to TASH from September 2014 to August 2018 were included in this study. Institutional ethical clearance for this study was received. Medical records were manually reviewed. Data collected include: presenting symptoms and signs, type (s) of seizure, history of previous seizure/epilepsy, cause (s) of seizure, identified comorbidities, investigations done (complete blood count, the most recent CD4 count, CSF analysis, imaging findings, serum antiepileptic drug level and reports of electroencephalography), drug reactions, treatment and out come at hospital discharge. Medical records with inadequate data to verify the diagnosis of seizure were excluded from the study. Patient data were given a code number to assure confidentiality.

International League Against Epilepsy method of seizure classification was used [15]. Status epilepticus was defined as continuous seizure activity lasting 30 min or more, or intermittent seizure activity lasting 30 min or more during which consciousness is not regained [16]. Preexisting history of epilepsy was defined as two or more seizures in a life time. The clinical staging of HIV-infection was based on WHO staging system [17]. The diagnosis of HIV-infection related diseases [cerebral toxoplasmosis, tuberculous meningitis, cryptococcal meningitis, bacterial meningitis, neurosyphilis, primary central nervous system (CNS) lymphoma, progressive multifocal leukoencephalopathy (PML) and HIV encephalopathy] was based on diagnostic criteria used in a previous study[8].

The data was analysed using SPSS 13.0 for Windows (SPSS, Chicago IL, USA). Logistic regression was used to assess each possible prognostic factor. Odds ratios and significant levels were calculated along with 95% confidence interval. A p-value of less than 0.05 was considered significant. A multivariate logistic regression analysis was performed to determine which prognostic factors, when considered together, were the best predictors of hospital death.

## Results

**Fig. 1:**
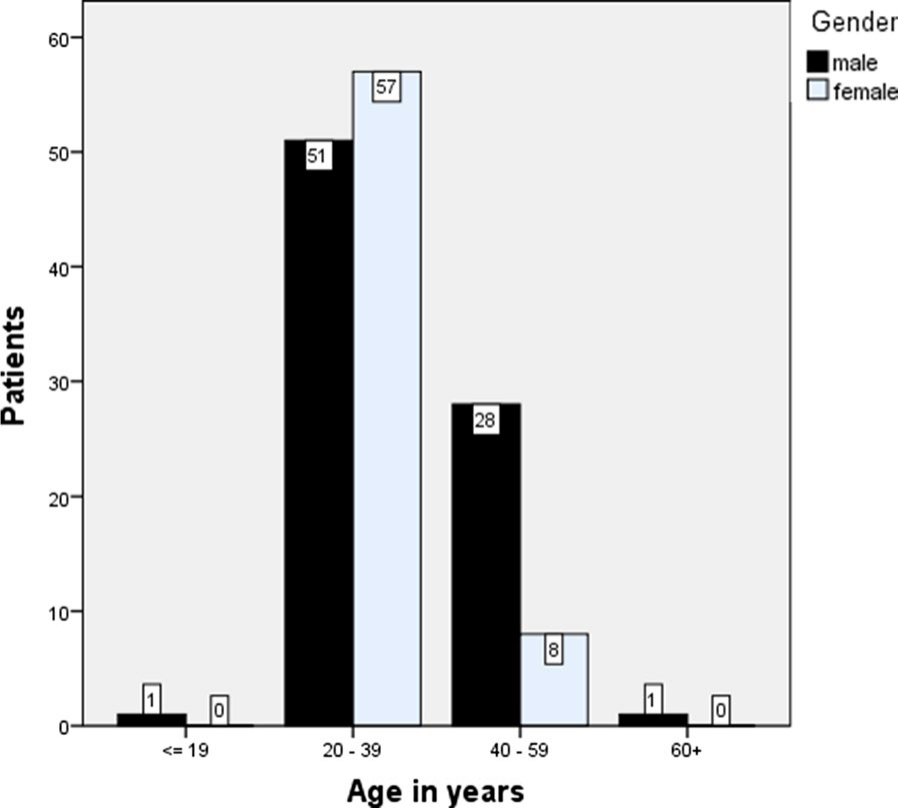
Age and sex distribution of 146 HIV infected patients with seizure admitted to Tikur Anbessa Specialized Hospital

Data from 146 HIV infected patients with seizure were analyzed; their sociodemographic and medical characteristics are shown in Fig. 1 and Table 1.

**Table 1:** Clinical profile of HIV-infected patients with seizure by outcome status, n = 146

	Total (n = 146)	Dead (n = 77)	Alive (n = 69)
Sex			
Male	81 (55.5%)	42 (51.9%)	39 (48.1%)
Female	65 (44.5%)	35 (53.8%)	30 (46.2%)
Age			
Mean [SD]	34[8.4]	33.78[8.6]	34.3[8.3]
Median	33	32	34
Range	51	41	45
Seizure type			
Simple focal	16 (11%)	11 (68.8%)	5 (31.3%)
FWSG	29 (19.9%)	12 (41.4%)	17 (58.6%)
Prim gener	101 (69.2%)	54 (53.5%)	47 (46.5%)
Status epilepticus			
Yes	26 (17.8%)	12 (46.2%)	14 (53.8%)
No	120 (82.2%)	65 (54.2%)	55 (45.8%)
Level of consciousness at initial evaluation			
Alert	35 (24%)	9 (25.7%)	26 (74.3%)
Confused/stuporous	61 (41.8%)	35 (57.4%)	26 (42.6%)
Comatose	50 (34.2%)	33 (66%)	17 (34%)
GCS at initial evaluation			
≥ 13	13 (8.9%)	5 (38.5%)	8 (61.5%)
9–12	26 (17.8%)	16 (61.5%)	10 (38.5%)
≤ 8	48 (32.9%)	33 (68.8%)	15 (31.3%)
Unknown	59 (40.4%)	23 (39%)	36 (61%)

*FWSG* focal with secondary generalization, *prim gener* primarily generalized, *GCS* Glasgow coma scale

Majority of the patients (80.8%) were from Addis Ababa. Most patients (93.8%) were admitted to general medical ward and 6.2% were admitted to intensive care unit (ICU). The mean hospital stay (± SD) was 23.3 ± 15.9 days (range, 1–76 days). The diagnosis of HIV-infection was made before current hospital admission in 31.5% and after current admission in 68.5%. Duration since the diagnosis of HIV-infection ranged from 7 days to 5 years (median 6 months).

The clinical presentation of patients is shown in Table 2. The initial presenting symptom was neurological in 131 (95.8%) patients and 21 (14.4%) patients presented with seizure as initial manifestation. The mean duration of initial symptom before presentation was 44.2 days (median = **21**, range 1 day to 18.3 months). Almost all patients (98.6%) had stage 4 HIV infection at presentation. Duration of first seizure at presentation varies from 30 min to a year (mean 22 days, median 5 days). All patients had more than two episodes of seizure. In 84 (57.5%) patients, seizure started before current hospital admission while in 62 (42.5%) patients the first seizure occurred after current admission. In patients with seizure onset before hospital admission, the duration of seizure was ≤ 1 day in 18%, ≤ 1 week in 64%, ≤ 2 weeks in 80%, ≤ 1 month in 88% and ≤ 3 months in 95% of the patients. The clinical seizure types were generalized tonic–clonic seizure (GTCS) in 101 (69.2%), focal motor with secondary generalization (FMWSG) in 29 (19.9%) and simple focal motor in 16 (11%) patients. The causes of seizure in relation to outcome status are shown in Table 3. Status epilepticus was present in 26 (17.8%) patients. The duration of status epilepticus at presentation ranged from 2 h to 7 days (mean = 31.1 h, median = 24 h). The status epilepticus started before admission in 24 patients and after admission in 2 patients. It was generalized convulsive type of status epilepticus in 24 (92.3%) patients and epilepsia partialis continua in 2 patients. The causes of status epilepticus were infectious in 25 (96%) of patients and metabolic in 1 patient (hyperkalemia and uremic encephalopathy). Identified infectious causes include cerebral toxoplasmosis (n = 16), tuberculous meningitis (n = 4), cryptococcal meningitis (n = 2), PML (n = 2) and HIV encephalopathy (n = 1). Complications of status epilepticus were present in 13 (50%) patients (aspiration pneumonia n = 12, acute renal failure n = 1).

**Table 2:** Clinical presentation of 146 patients with HIV infection and seizure presented to Tikur Anbessa Specialize Hospital

Symptom/sign	Frequency (n)	Percentage (%)
Headache	111	76
Change in mentation	111	76
Fever	96	65.8
Focal deficit		
All types	75	51.4
Hemiparesis/plegia	44	30.1
MCN* deficit	9	6.2
Paraparesis/plegia	3	2
Monoparesis/plegia	3	2
Quadriparesis/plegia	2	1.4
Triparesis/plegia	1	0.7
Meningeal irritation sign	55	37.7
Papilledema	17	11.6
hemiballismus	2	1.4

*MCN* multiple cranial nerve deficit

**Table 3:** The causes of seizure by outcome in 146 HIV infected patients

Cause of seizure	Total (146)	Dead (n = 77, 52.7%)	Alive (n = 69,47.3%)
Cerebral toxoplasmosis	67	29 (43.3%)	38 (56.7%)
Tuberculous meningitis	52	34 (65.4%)	18 (34.6%)
Cryptococcal meningitis	20	13 (65%)	7 (35%)
PML	7	3 (42.9%)	4 (57.1%)
neurosyphilis	4	2 (50%)	2 (50%)
Bacterial meningitis	3	1 (33.3%)	2 (66.7%)
stroke	3	2 (66.7%)	1 (33.3%)
Uremic encephalopathy	2	2 (100%)	0
Unknown (except HIV infection)	2	0	2 (100%)
Others	3	2 (66.7%)	1 (33.3%)

Others: HIV encephalopathy/dementia = 1, neurocysticercosis n = 1, primary CNS lymphoma n = 1.

Note that more than one possible cause of seizure was possible in some patients

Altered mental status was found in 111 (76%) of patients and 50 (34.2%) patients presented with coma. Glasgow coma scale (GCS) was documented in 87 (59.6%) patients and 48 (55.2%) had ≤ 8, 26 (29.9%) had 9 to 12 and 13 (14.9%) had ≥ 13.

CD4 count per mm3 was obtained in 96 (65.8%) patients and the mean was 77 ± 85 (median = 52, range 1 to 550). Lumbar puncture was done in 87 (59.6%) patients. The mean and median CSF cell count per mm3 was 177.26 and 1.5, respectively (range 0 to 2500). CSF VDRL test was performed in 72 patients and 4 were reactive. India ink was done in 75 patients and 20 (26.7%) were positive.

Brain imaging with CT-scan was performed for 76 (50.1%) patients and 3 patients were additionally evaluated with brain MRI. Brain imaging showed mass lesion in 43 (56.6%), normal findings in 19 (25%), multiple white matter hypodense lesions in 7 (9.2%), meningeal enhancement (n = 4), brain atrophy (n = 2) and hydrocephalus (n = 1).

Hematologic findings were: leucopenia in 46 (31.5%), anemia in 81 (55.5%) and thrombocytopenia in 54 (37%) patients. Erythrocyte sedimentation rate was obtained in 99 (67.8%) patients and in 96 (97%) patients it was 35 mm per hour or more (mean = 92.32, median = 94). One patient underwent electroencephalography (EEG) and it was normal. Serum antiepileptic medication level was determined in one patient and it showed low phenytoin level.

Comorbidities identified were: oral thrush (n = 25), herpes zoster (n = 21),hypertension (n = 6), bleeding diathesis (5),drug induced hepatitis (n = 5), hypokalemia (n = 4), pneumonia (n = 4),chronic diarrhea (n = 4), diabetes mellitus (n = 4), congestive heart failure (n = 4),deep venous thrombosis (n = 3), pneumocystis carinii pneumonia (n = 2)and bed sore (n = 4).

Antiepileptic drugs (AED) were given to 135 (92.5%) after hospital admission and 11 (7.5%) patients were on antiepileptic medications at admission. The AED used were: phenytoin in 137 (93.8%), Phenobarbital (n = 15), carbamazepine (n = 5), and valproate (n = 3). Of the 69 patients who were discharged alive, 56 (81.2%) were continued on the AED and in 13 (18.8%) patients these were discontinued. Antiretroviral therapy (ART), which includes lamivudine (n = 62), efavirenz (n = 51), tenofovir (n = 37), zidovidine (n = 25) and nevirapine (n = 11), was started before hospital admission in 24 (16.4%) and after admission in 38 (26%) patients. No ART was given to 84 (57.5%) patients. The duration of ART before admission ranges from 1 to 30 months (mean = 6.09, median = 3). Prophylactic cotrimoxazole was started in 65 (44.5%) patients (before admission = 10, after admission = 55).

Treatment out comes at hospital discharge were: 77 (52.7%) patients died, 60 (41.1%) improved, 7 (4.8%) deteriorated without death and 2 remained in the same clinical condition. In most patients, the immediate cause of death was attributed to the underlying disorder and seizure was not documented as an immediate cause of death.

## Discussion

The objectives of this hospital-based retrospective study were to assess the clinical presentation, causes and treatment outcome of HIV-infected patients with seizure who presented to TASH which is the largest hospital in Ethiopia. The number of male (55.5%) and female were comparable (OR = 0.923, 95% CI = 0.565–2.07) which is similar to other study [3]. Unlike this study male predominance was reported in other studies [4–7, 10, 18, 19]. The mean hospital stay in days was 23.3 (± 15.9) which is longer compared to other study [10] which is partly due to the advanced stage of HIV infection in most of the patients. The mean age of patients was 34 ± 8.4 years. Majority (88%) of them were below the age of 45 years which is similar to other studies [4–7, 10]. This might be partly due to the fact that the Ethiopian population is young [20] where 86.2% were below the age of 45 years in 2007.

Majority (69%) of the patients had primarily generalized type of seizure like in most other studies [4–7, 9, 11–13]. Even thought almost all patients (98.6%) had stage 4 HIV infections at presentation, in the majority (68.5%) the diagnosis of HIV infection was made after current hospital admission. Similar to other studies [4, 9], seizure occurred in patients with advanced HIV infection in these patients. The initial presenting symptom/sign was neurological in almost all patients ( 96%) patients: headache (76%), change in mentation (76%), focal deficit (51.%), meningeal irritation signs (37.7%) and papilledema (1.6%) which is consistent with other studies [5–8] in which the incidence of seizure is common in patients with neurological complications of HIV infection. Seizure as an initial manifestation was uncommon in this study (14%) similar to other studies [7, 14, 19]. In most patients, seizure was a recent onset at admission. It either started after current hospital admission (42.5%) or within 3 months prior to admission (52.5%). This indicates that most seizures were related to recent neurological complications of HIV infection.

The common causes of seizure were cerebral toxoplasmosis (46%), tuberculos meningitis (35.6%) and cryptoccocal meningitis (13.7%) which is similar to most other studies [6, 7, 9, 11–13]. Toxic-metabolic (59%) and HIV encephalopathy (22%) were the commonest causes of seizure in other studies [4, 5], respectively. HIV encephalopathy which is a common cause of seizure in developed countries [5, 9, 10, 14] was rare in this study like most other studies done in developing countries [7, 12, 13]. This may be partly explained by the fact that early and effective treatment of HIV-infection is not optimal in developing countries; hence, HIV-infected patients have a higher incidence of CNS opportunistic infections.

Status epilepticus occurred in 18% of the patients which is similar to some studies [4, 6, 14] but higher compared to other studies [7, 19]. The cause of status epilepticus was CNS infection in almost all patients which is related to the advanced stage of HIV infection and low CD4 count (mean: 77 ± 85 per mm^3^). The mean CD4 count in this study was lower compared to some studies [4, 5] and higher compared to other studies [9, 10]. The most common abnormality identified on brain imaging (done in 52%) was mass lesion (n = 43, 57%) which was due to cerebral toxoplasmosis in almost all patients. EEG and serum AED serum level was done in one patient each which indicates the limitations of diagnostic facilities in the hospital.

The AED of choice in patients with HIV infected patients is levetriracetam and where the newer AED are not available, valproic acid may be the treatment of choice [21]. In contrast to this recommendation, 94% of the patients were treated with phenytoin. Phenytoin drug reaction was not reported in these patients and was well tolerated as seen in other studies [4, 13]. In contrast to this study, phenytoin drug reaction was seen in 25% and 14% of patients in other studies [6, 14], respectively. Other authors [22, 23] also reported that even though phenytoin is the most widely prescribed anticonvulsant, hypersensitivity reactions are common. Even though almost all patients were having advanced HIV infection (stage 4), only 16% were on ART during current admission which indicates early diagnosis and timely treatment of HIV infection is suboptimal in this region.

The over-all mortality was 52.7% which is higher compared to other studies [4, 7, 18] which reported mortality of 22% to 47%. Even though address from Addis Ababa (the capital city where the hospital is found), seizure started after admission, change in mentation at initial evaluation and comatose at initial evaluation were predictors of mortality in the univariate analysis (Table 4), only address from Addis Ababa and change in mentation at initial evaluation were independent predictors of mortality. The higher mortality in patients from Addis Ababa was unexpected finding. This might be partly due to the fact that patients who were critically ill were not able to travel to Addis Ababa from other parts of the country. Seizure as an immediate cause of death was not reported in this study and there was no relationship between status epilepticus and mortality similar to other study [4]. In contrast to this, other authors [24] reported that status epilepticus was a predictor of mortality. Unlike a study done in Zambia [18] which reported women to have a higher mortality, gender was not associated with mortality in this study.

**Table 4:** Risk factor for case-fatality: logistic regression analysis

Factor	n	%	Unadjusted OR (95% CI)	Adjusted OR (95% CI)
Age (year)				
≥ 40	20	54.1	1.00	1.00
< 40	57	52.3	0.932 (0.441–1.968)	0.67o (0.274–1.641)
Sex				
Female	35	53.8	1.00	1.00
Male	42	51.9	0.923 (0.480–1.775)	0.922 (0.429–1.983)
Address				
Outside Addis Ababa	10	35.7	1.00	1.00
Addis Ababa	67	56.8	2.365 (1.006–5.558)	3.428 (1.240–9.479)
Admitted to				
Intensive care unit	3	33.3	1.00	1.00
Ward	74	54	2.349 (0.564–9.778)	2.818 (0.506–15.686)
Seizure started				
Before admission	37	44	1.00	1.00
After admission	40	64.5	2.310 (1.175–4.538)	2.017 (0.889–4.575)
Status epilepticus				
No	65	54.2	1.00	1.00
Yes	12	46.2	0.725 (0.310–1.698)	0.526 (0.156–1.781)
Change in mentation at initial evaluation				
No	9	25.7	1.00	1.00
Yes	68	61.3	4.568 (1.955–10.675)	3.981 (1.475–10.749)
Comatose at initial evaluation				
No	44	45.8	1.00	1.00
Yes	33	66	2.294 (1.128–4.664)	2.035 (0.828–5.005)
CD4 count				
≥ 200	32	58.2	1.00	1.00
< 200	45	49.5	0.703 (0.358–1.381)	0.730 (0.313–1.703)
Antiretroviral therapy				
No	48	57	1.00	1.00
Yes	29	46.8	0.659 (0.341–1.275)	0.632 (0.268–1.493)

This study had several limitations. During chart review missing information may occur. The retrospective nature of the study may cause under-ascertainment of cases. No patient with complex partial seizure was reported. Given the advanced stage of illness, patients with subtle focal seizure may be missed. Patients who were treated as outpatient only were not included in this study. With limited service of EEG, patients with non-convulsive seizure may not be identified. With limited access to brain imaging, biopsy and CSF analysis, etiological diagnosis may be difficult in some patients. Since TASH is a tertiary referral hospital, chance of selectively admitting critically ill patients is high.

To improve the high morbidity and mortality observed in patients with HIV-infection presented with seizure, prevention of HIV-infection, early diagnosis and treatment, early identification and treatment of neurological complications of HIV-infection are recommended. Improving diagnostic facilities (brain imaging, CSF analysis, EEG and biopsy) may improve treatment outcome. Replacing enzyme-inducer AEDs with non-enzyme-inducers may help improve effectiveness of ART.

## Conclusion

The diagnosis of HIV-infection was made after current hospital admission in majority of patients, almost all were in stage IV HIV-infection with very low CD 4 count, case-fatality was higher than most other studies and change in sensorium was an independent predictor of mortality. To improve the high mortality, HIV prevention, early diagnosis and treatment, early identification and treatment of neurological complications of HIV-infection, improving diagnostic facilities (brain imaging, EEG and CSF analysis) and improving access to non-enzyme inducing AEDs are recommended.

## Data Availability

The data used during the current study are not publicly available but are available from the author on reasonable request.

## Abbreviations

AED: Antiepleptic drugs
ART: Antiretroviral therapy
CNS: Central nervous system
CSF: Cerebrospinal fluid
CT: Computerized tomography
EEG: Electroencephalography
FMWSG: Focal motor with secondary generalization
GCS: Glasgow coma scale
GTCS: Generalized tonic–clonic seizure
ICU: Intensive care unit
MRI: Magnetic resonance imaging
PML: Progressive multifocal leukoencphalopathy
TASH: Tikur Anbessa Specialized Hospital
VDRL: Venereal Disease Research Laboratory
